# Radiolabeled Probes from Derivatives of Natural Compounds Used in Nuclear Medicine

**DOI:** 10.3390/molecules29174260

**Published:** 2024-09-08

**Authors:** Giuseppe Tesse, Anna Tolomeo, Barbara De Filippis, Letizia Giampietro

**Affiliations:** 1 Radiopharma Division, ITEL Telecomunicazioni s.r.l., 70037 Ruvo di Puglia, BA, Italy; g.tesse@itelte.it (G.T.); a.tolomeo@itelte.it (A.T.); 2 Department of Pharmacy, Università degli Studi G. d’Annunzio, 66100 Chieti, CH, Italy; barbara.defilippis@unich.it

**Keywords:** radiopharmaceutical, natural compounds, nuclear medicine, cancer, Alzheimer’s disease, curcumin, stilbene, chalcone, benzofuran

## Abstract

Natural compounds are important precursors for the synthesis of new drugs. The development of novel molecules that are useful for various diseases is the main goal of researchers, especially for the diagnosis and treatment of many diseases. Some pathologies need to be treated with radiopharmaceuticals, and, for this reason, radiopharmaceuticals that use the radiolabeling of natural derivates molecules are arousing more and more interest. Radiopharmaceuticals can be used for both diagnostic and therapeutic purposes depending on the radionuclide. β^+^- and gamma-emitting radionuclides are used for diagnostic use for PET or SPECT imaging techniques, while α- and β^−^-emitting radionuclides are used for in metabolic radiotherapy. Based on these assumptions, the purpose of this review is to highlight the studies carried out in the last ten years, to search for potentially useful radiopharmaceuticals for nuclear medicine that use molecules of natural origin as lead structures. In this context, the main radiolabeled compounds containing natural products as scaffolds are analyzed, in particular curcumin, stilbene, chalcone, and benzofuran. Studies on structural and chemical modifications are emphasized in order to obtain a collection of potential radiopharmaceuticals that exploit the biological properties of molecules of natural origin. The radionuclides used to label these compounds are ^68^Ga, ^44^Sc, ^18^F, ^64^Cu, ^99m^Tc, and ^125^I for diagnostic imaging.

## 1. Introduction

Nuclear medicine is a branch of medicine that uses radiopharmaceuticals for its purposes [1]. A radiopharmaceutical consists of a targeting moiety and a radionuclide. Chemical linkers are used to stabilize the links between the targeting structures and the radionuclides, because the targeting moieties have the aim of driving the radionuclide using the body’s chemical and biological processes. For this reason, the targeting moieties must have a high selectivity and specificity towards their targets. Transporters, enzymes, certain receptors, or antigens are examples of these target sites. The physicochemical conditions (pH, light, temperature, etc.) for their stability must be properly set for the manufacturing and storage of radiopharmaceuticals [2].

Radionuclides decay has different emission of particles or electromagnetic radiation (positrons, gamma rays, etc.). These emissions are important for the selection of a radionuclide for imaging or therapy in nuclear medicine [3]. The most important nuclear imaging techniques are single-photon emission computed tomography (SPECT) and positron-emission tomography (PET), in which radiopharmaceuticals are administered intravenously [3]. PET is a noninvasive imaging methodology in which the administration of compounds labeled with positron-emitting radioisotopes (radiotracers or radioligands) are substrates that localize the particular targets of diseases [4]. The most commonly used PET tracers are radiolabeled with fluorine-18 (^18^F) and they are used to detect various diseases, such as cancer, brain pathologies, heart diseases, and bone lesions. SPECT, on the other hand, is a technique that uses gamma-emitting radionuclides such as iodine-123 (^123^I), technetium-99m (^99m^Tc), xenon-133 (^133^Xe), thallium-201 (^201^Tl), and indium-111 (^111^In), and these radioligands are used to diagnose seizures, strokes, bone diseases, and infections [2].

Radiopharmaceuticals play an important role in the diagnosis and treatment of many diseases. The use of a lot of radiopharmaceuticals can be an important and effective approach in oncology when other standard therapeutic strategies fail to achieve positive results [5,6,7]. The use of nuclear radiotherapy is important to deliver the therapeutic radiation to the target in the body [8,9,10]. For this reason, nowadays, many active compounds are studied as potential radiopharmaceuticals, including organic molecules, nanoparticles, peptides, and monoclonal antibodies [5,6,7].

The radionuclides used for therapy emit alpha (α) particles (50–230 keV/µm) and beta (β^−^) particles (0.2 keV/µm) [11,12,13,14]. These particles have a higher linear energy transfer (LET) than the gamma rays or positrons used in imaging [12,15]. The affected cells are destroyed directly and indirectly. To maximize targeted cell killing and minimize the interaction of ionization with healthy cells, it is important to evaluate both the energy deposited in the cells and the distance traveled by the particles.

The most common beta particle is iodine-131 (^131^I), which is used especially for the treatment of thyroid cancer. Yttrium-90 (^90^Y) and lutetium-177 (^177^Lu) are other beta particle emitters used to label monoclonal antibodies. These radiolabeled monoclonal antibodies are important for the therapy of ovarian and hematologic cancers (^90^Y) and for the therapy of prostate cancer (^177^Lu).

Target α particle therapy (TAT) uses alpha radiation that creates double strand breaks (DSBs) and a lot of clusters in the DNA of the target cells. The radionuclide used in TAT is radium-223 (^223^Ra), which is important for relieving bone pain in prostate and breast cancer patients [16]. Another alpha particle emitter is ^225^Ac, which can be used to treat neuroendocrine tumors [17].

Compounds derived from natural products such as oils and medicinal plants are playing an increasingly important role in the search for new selective molecules to treat various diseases such as cancer, neurodegenerative diseases, inflammation, and microbial and fungal infections [18]. The negative aspects of natural compounds are their high molecular weight, poor stability, and low solubility [19]. These natural molecules are the target of drug discovery because they exhibit different pharmacological activities [20]. Two components extracted and purified from *Nuxia oppositofilia*, namely, 3-oxolupenal and katononic acid, were studied as inhibitors of α-amylase and α-glucosidase, showing antidiabetic and antioxidant activity [21]. Stilbenes showed excellent inhibitory potential against α-glucosidase [22]. The garlic extracts were studied, and they showed inhibitory activity on dipeptidyl peptidase-4, the serine protease that regulates glucose metabolism and catalyzes the degradation of glucagon-like peptides (GLP-1 and GLP-2) and the glucose-dependent insulin releasing polypeptide GIP [23].

Another important pharmacological target of natural compounds are the protein kinases. These enzymes regulate important cellular processes including proliferation, differentiation, apoptosis, and cell metabolism. The malfunction of these enzymes is the cause of many diseases such as cancer, neurological disorders, and autoimmune diseases [19]. In nature, there are many molecules that are able to inhibit the activity and signaling of Ras proteins. These proteins are important to maintain the growth of different types of cancer cells. For these reasons, Ras inhibitors could be important to decrease the growth of cancer [24].

Natural compounds that are present in many herbs, vegetables, and fruits possess antioxidant activity and are used to prevent inflammatory diseases and fight cancer due to their prooxidant and antioxidant properties, because they have many anticancer properties such as preventing the formation of reactive oxygen species (ROS), reducing angiogenesis of cancer cells, and enhancing the DNA oxidation of tumor cells [25]. ROS are oxygen-containing molecules and radicals that are produced in the mitochondria during the oxidative metabolism or in response to external stimuli (such as xenobiotics or bacterial invasion) [26].

Molecules of natural origin have also been studied for their ability to decrease the cellular aging process, which involves a loss of function at the cellular, tissue, and organ levels. This biological process is characterized by oxidative stress, in which there is an increase in ROS and a decrease in autophagy, which is responsible for the elimination of damaged cells. The increased proinflammatory stimuli and the decreased homeostasis of the anti-inflammatory response are the causes of molecular and cellular oxidative damages in all tissues [27].

These studies demonstrate the importance of molecules from nature for the development of targeted therapies for the treatment of numerous diseases.

The development of new radiopharmaceuticals is dominated by molecules of synthetic origin. Nevertheless, the development of radiopharmaceuticals from natural products is studied, because natural bioactive molecules are showing promising applications in the field of nuclear medicine.

Bioactive precursors of radiopharmaceutical candidates must have an appropriate site for labeling with radionuclides. For this reason, it is important to modify some chemical groups, such as the conversion of a specific group into a functional moiety that enables conjugation with a radionuclide, the protection and deprotection of nucleophilic groups to avoid unwanted side reactions, and the addition of a chelator or linker to prevent steric impact on binding to the receptor. A lot of radiopharmaceuticals derived from naturally occurring bioactive compounds have been synthesized and developed, and they have demonstrated promising applications in the field of nuclear medicine, despite the limited use of natural products as a template for the production of novel radiopharmaceuticals. In this regard, slowness in isolation and difficulty in structure elucidation are frequently linked to the complexities of natural product chemistry [28].

The aim of this review is to highlight the development of radiopharmaceuticals using the radiolabeling of natural derivate molecules, with the aim to obtain new compounds with different pharmacological activities.

## 2. Curcumin Derivates

**Figure 1:**
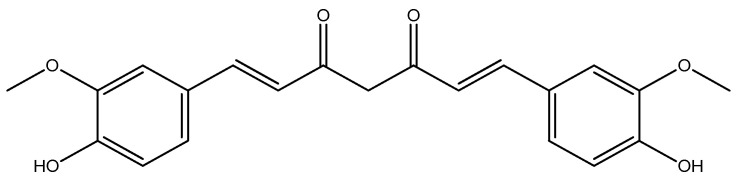
Chemical structure of curcumin.

Curcumin (CUR) [(*E*,*E*)-1,7-bis(4-hydroxy-3-methoxyphenyl)-1,6-heptadiene-3,5-dione] (Figure 1) is an active molecule of turmeric, a yellow compound found in the rhizomes of *Curcuma longa* L., a plant of the ginger family.

CUR can be extracted and purified from the dried rhizomes using traditional or green techniques. This component and its derivates are important for their antioxidant, anti-inflammatory, and anticancer effects [29].

CUR was demonstrated to inhibit the proliferation of many types of cancer cells; indeed, it is important in fighting different types of cancer, such as cancer of the breast, lung, blood, and digestive system [30]. Moreover, CUR and its derivates express anticancer activity, suppressing the proliferation of these tumor cell lines, modifying the deregulated cell cycle through p53-dependent, p53-independent, and cyclin-dependent pathways. There are a lot of clinical trials that study the safety and efficacy of CUR in other types of cancer (pancreatic cancer, multiple myeloma, colon cancer, and myelodysplastic syndromes) [3].

Some studies established that CUR showed a fast metabolism and poor absorption. For this reason, the development of CUR analogues as drug probes and imaging tracers is important in research [31].

Dietary CUR lowered the presence of β-amyloid plaque in the hippocampus and cortex regions of APPsw Tg2576 transgenic mouse brain sections [9]. For these important features of CUR, in the nuclear medicine field, radiolabeled CUR derivates have been studied as potential radiotracers for the early diagnosis of Alzheimer’s disease (AD) and cancer [1].

**Figure 2:**
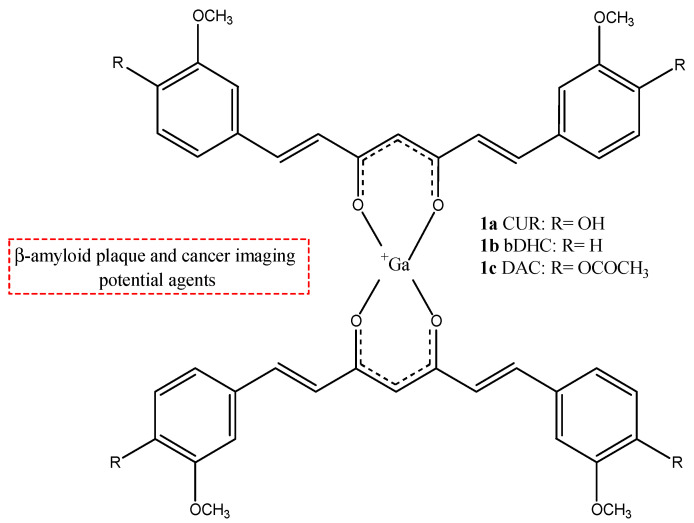
Chemical structures of ^68^Ga-labeled complexes with curcuminoids **1a**–**c**.

CUR is a symmetric molecule and has two *o*-methoxy phenolic aromatic rings connected by an unsaturated, seven-membered carbon chain with a β-diketo moiety. It is important for its keto-enol tautomeric equilibrium, which can be in enolic or di-keto form depending on the pH and the solvent. For this chemical feature, CUR is able to form stable complexes with metals. Nuclear medicine can use this feature to form new radiotracers for PET or SPECT, forming CUR complexes with some radionuclides. The most frequently used radionuclide that is used to form some radioprobe complexes of CUR derivates is gallium-68 (^68^Ga). This radionuclide is used in PET clinicals for its commercial availability generators (^68^Ge/^68^Ga generator), thus avoiding the need to have a cyclotron on site [29]. The bioavailability of CUR is limited because it has a low solubility and stability in aqueous media. This problem may be resolved by modifying the structure of CUR itself to increase its chemical stability and solubility but preserving its beneficial properties. In fact, the α,β-diketone group of the CUR structure is subjected to a pH- and solvent-dependent keto-enol tautomerism that influences the CUR metal chelation capability [32]. A lot of curcumin derivatives have been studied, improving efficacy and activity and enhancing bioavailability [28]. ^68^Ga-labeled complexes with CUR (**1a**) and two curcuminoids, namely, diacetyl-curcumin (DAC) (**1b**) and bis(dihydroxy)curcumin (bDHC) (**1c**) (Figure 2), were synthesized and these showed both enhanced chemical stability, with a radiochemical yield greater than 95% at pH 5. Their affinity for synthetic β-amyloid fibrils and their uptake by A549 lung cancer cells were studied to show their potential use in the diagnostic fields in nuclear medicine [32].

The uptake of complexes **1a**–**c** by different tumor cell lines of different isotypes was studied and compared to the uptake of the same compounds in normal human lymphocytes: complex **1c** showed a preferred uptake in HT29 colorectal cancer cell and K562 lymphoma cell line with respect to normal human lymphocytes, hinting at a possible application of these compounds as future radiotracers for cancer [30].

**Figure 3:**
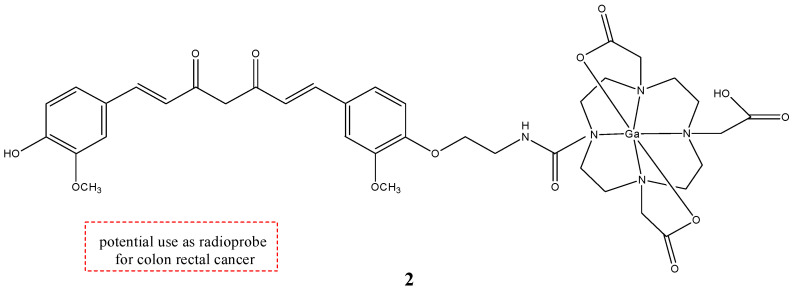
Chemical structure of ^68^Ga-DOTA-C21, **2**.

The mechanism of entrance of CUR and its derivates inside the cell is not known thus far, but CUR was investigated as a potential novel vitamin D receptor (VDR) ligand. CUR binds directly to and activates VDR, playing a role in colon cancer chemoprevention [33]. Complexes **1a**–**c** have a good stability in phosphate buffer solution (PBS) and human serum; however, they are rapidly degraded in human blood. For this reason, a new gallium-68-labeled CUR derivate (**2**), named ^68^Ga-DOTA-C21, was also studied (Figure 3) [34].

In this metal complex, the CUR is linked to an efficient gallium-68 chelator 1,4,7,10-tetraazacyclododecane-1,4,7,10-tetraacetic acid (DOTA). DOTA was linked through an aminoethyl spacer to one of the phenol groups of the curcumin. It was shown that with this chelator, the new bioconjugate has better water solubility and higher stability in physiological conditions than the other curcuminoid complexes. This new gallium complex **2** was investigated to study its possible use as probe for colon rectal cancer (CRC). For this reason, the uptake, internalization, and efflux of complex **2** were studied in HT29 colorectal cancer cell lines. Complex **2** showed a time-dependent cellular accumulation in HT29 in vitro. To test the uptake, HT29 cells were incubated with an excess of calcitriol, the natural ligand of VDR. Then, complex **2** was added to these cells. The uptake of gallium complex is similar with or without the preincubation of the cells with calcitriol. This underlines that VDR was not involved in the uptake of the gallium complex **2**. With regard to efflux studies, molecule **2** showed a slow externalization pattern with around 75% of the radiotracer that is within the cell after 60 min of incubation. Biodistribution studies showed that **2** has both renal and hepatic clearance. Furthermore, no radioactivity was seen in the intracranial region, so it is possible that the complex does not cross the blood–brain barrier (BBB). The blood radioactivity was high, suggesting that this complex bound to the blood constituents as serum albumin [34].

**Figure 4:**
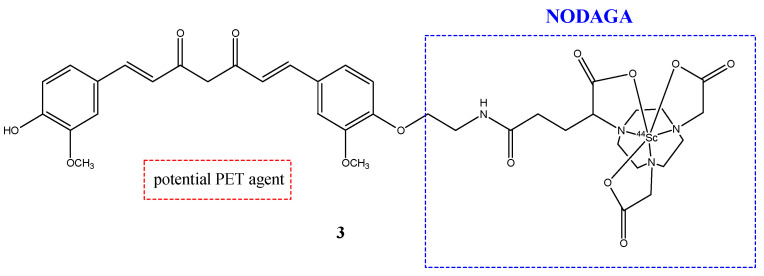
Chemical structure of NODAGA-C21, **3**.

**Figure 5:**
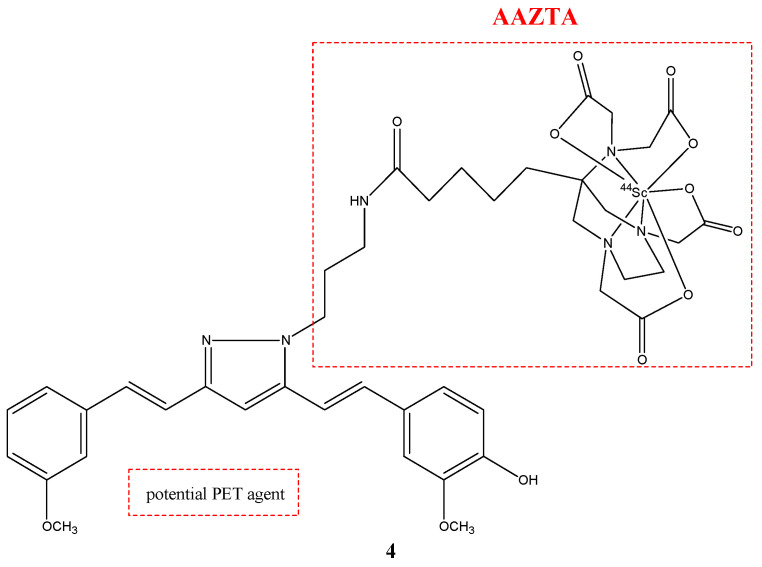
Chemical structure of AAZTA-PC21 (**4**).

Scandium-44, as well as gallium-68, was used to study new possible radioprobes with curcuminoids derivates. Scandium-44 is a β^+^-emitting radionuclide and has a half-life four times longer than gallium-68 (3.97 h) [28]. Two curcuminoid complexes with scandium-44 were synthetized and studied. The first compound was NODAGA-C21 (**3**), in which the chelator was 1,4,7-triazacyclononane,1-glutaric acid-4,7-acetic acid (NODAGA) (Figure 4); while the second compound was AAZTA-C21 (**4**), which used 1,4-bis(carboxymethylperhydro-1,4-diazepine (AAZTA) (Figure 5) [30].

The curcuminoids **3** and **4** were also studied to improve their stability in physiological conditions. Lipophilicity is an important parameter to have some information about the distribution of radiopharmaceuticals in vivo. In fact, the radiopharmaceuticals should have a lower lipophilicity to have a reduced uptake in liver and intestines and a better biodistribution. For this reason, a protic bifunctional chelator was introduced to decrease the lipophilicity of the derivates more than the curcumin, which has a high lipophilicity [29]. From these studies, the complexation of NODAGA-C21 with scandium-44 was quite slow and poorly reproducible (incorporation < 50% after 30 min of incubation at 95 °C), while the [^44^Sc]Sc-**4** complex was obtained after 10 min at room temperature and its stability was considerable (>90% after 8 h of incubation in PBS or human serum and around 75% and 60% after 2 and 8 h in human blood, respectively) [28].

**Figure 6:**
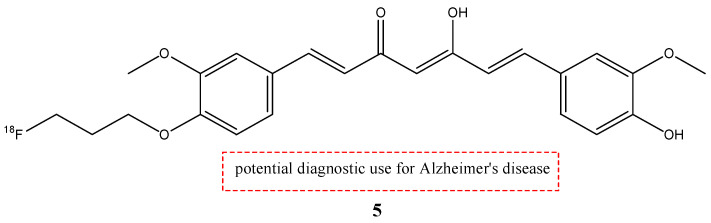
Chemical structure of [^18^F]FP-Cur (**5**).

Nowadays, the most used radionuclide for PET imaging is fluorine-18, positron-emitting radionuclide (E_β, max_ = 634 keV, 109.8 min) [28]. Ryu E.K. et al. synthesized one of the first ^18^F-labeled curcumin derivatives: (1*E*,4*Z*,6*E*)-1-(4-(3(-[^18^F]fluoropropoxy)-3-methoxyphenyl)-5-hydroxy-7-(4-hydroxy-3-methoxyphenyl)-hepta-1,4,6-trien-3-one (**5**) (fluoropropylcurcumin; FP-curcumin) (Figure 6) [35].

Curcumin derivative **5** showed excellent binding affinity (Ki = 0.07 nM) for Aβ (1–40) aggregates; in fact, the radioligand showed a high uptake in the hippocampus and cerebellum in transgenic mouse (Tg2576) brain sections. However, it showed an initial brain uptake that was low (0.52% ID/g at 2 min post-injection), because compound **5** has a rapid metabolism in the liver and in the intestinal wall, as curcumin.

**Figure 7:**
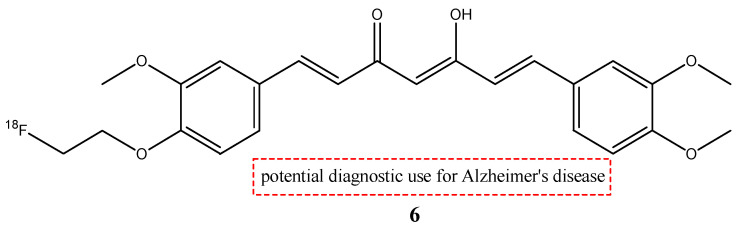
Chemical structure of [^18^F]FEM-Cur (**6**).

In their study, Ryu E.K. et al. synthesized another molecule: 1-(4-fluoroethyl)-7-(4′-methyl) curcumin (**6**, FEM-Cur) (Figure 7) [29,35].

This curcumin derivate has a high affinity for β-amyloid plaques, so it was chosen as a possible radioprobe for PET for Alzheimer’s disease. The biodistribution and the pharmacokinetics of compound **6** in normal mice were comparable to those of compound **5**. At 2 min post-injection, more radioactivity was accumulated in the lungs, liver, and spleen, but then rapidly decreased over time. On the contrary, the brain uptake of **6** was 2.7-fold higher than the uptake of **5** at 2 min post-injection (1.44% ID/g) and grew to 4.1-fold at 30 min post-injection (0.45% ID/g). From these results, the high lipophilicity of **6** was shown, and this feature allowed a higher BBB permeability. These features allow us to consider that molecule **6** could be a potential radiotracer for the imaging of β-amyloid plaques, but other studies are warranted [28].

**Figure 8:**
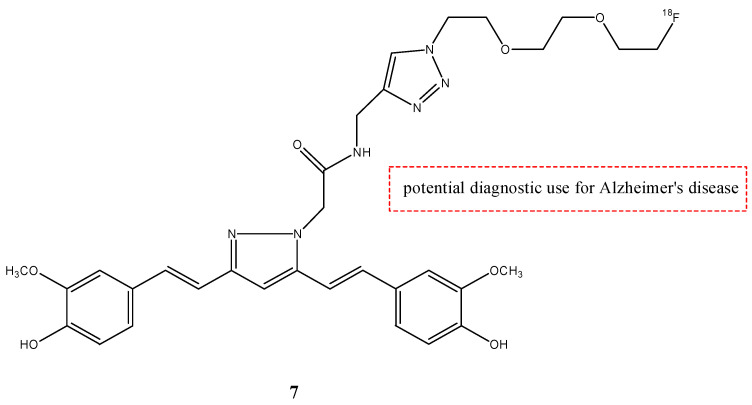
Chemical structure of compound **7**.

From these results, CUR had poor bioavailability and showed a rapid metabolism in the liver and intestinal walls [36]. The reason for this is the high reactivity of the keto-enol moiety that exhibits limited solubility in physiological media and a low stability in vivo. All the radiotracers based on curcumin show these limitations and a rapid metabolism. To avoid these problems, the keto-enol moiety was converted to a pyrazole ring in the research of Rokka et al. that synthetized the compound **7** (Figure 8) as a possible radiotracer for Alzheimer’s disease (AD) [33].

This curcumin derivate **7** was studied to evaluate its binding to β-amyloid plaques in vitro on brain cryosections of transgenic APP23 mice and wild-type mice [37]. Molecule **7** showed a specific accumulation on β-amyloid plaques in vitro, but it showed a very low brain accumulation in vivo with the same pharmacokinetics in the two animal models through a 60 min dynamic PET scan. Compound **7** showed a low stability in mice’s blood, with only 45% of intact radiotracer after 10 min of injection. The scientists arrived at the conclusion that low BBB penetration and low stability are significant obstacles to the successful detection of Aβ-amyloid accumulation in vivo, despite the fast blood clearance and encouraging in vitro results.

**Figure 9:**
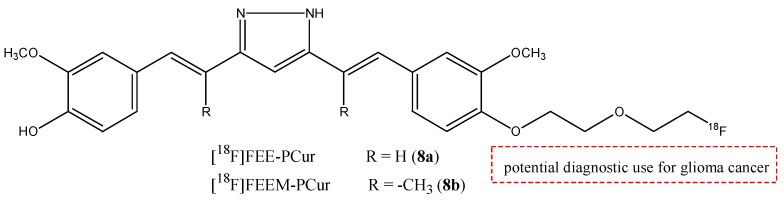
Chemical structures of [^18^F]FEE-PCur (**8a**) and [^18^F]FEEM-PCur (**8b**).

Other two curcumin derivates were studied as potential radiotracers for the visualization of the tumors by Shin et al. [38]. These molecules are [^18^F]FEE-PCur (**8a**) and [^18^F]FEEM-PCur (**8b**) (Figure 9) [38]. These pyrazole curcumins were studied to evaluate the antitumoral activity in C6 glioma cells. The radiotracers were injected in mice bearing C6 glioma xenografts. Both the two molecules demonstrated high accumulation in the intestines, but **8b** showed higher tumor uptake than **8a** at 65 min post-injection (3.2% and 0.98% ID/g, respectively); **8a** showed higher uptake in the small intestine, indicating a more rapid clearance. Further studies are needed to study the potential use of these two molecules as radioprobes for PET imaging for cancer [28].

## 3. Stilbene Derivates

**Figure 10:**
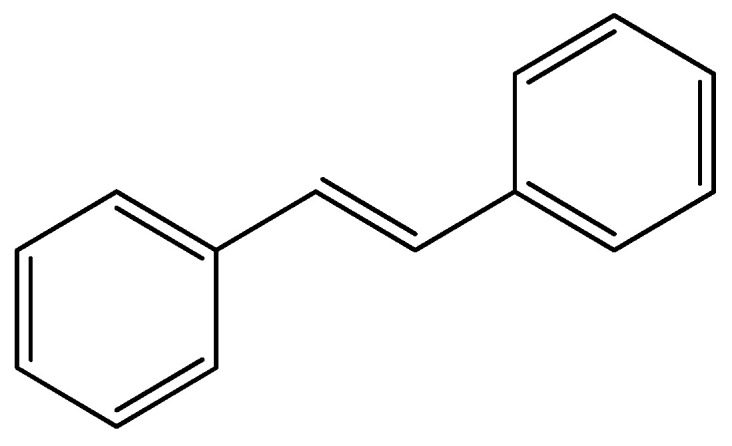
Chemical structures of stilbene.

Nowadays, stilbene-based derivate molecules are studied for their activity in cancer prevention [39,40]. Stilbene is a molecule lead characterized by two aromatic rings linked by an ethylene bridge (Figure 10).

Stilbenoids are produced by a variety of plants, including blueberries, peanuts, and grapes, in response to physiological or stressful stimuli. Stilbenoids can have two configurations of the central double bond: *Z*-type and *E*-type. Stilbene derivates need to avoid the photoisomerization *Z*/*E* to improve their stability and their biological activity, and are studied for their variety of biological activity properties such as antioxidant [41,42,43], hypolipidemic [44,45], antiviral [46,47], anti-inflammatory [48], and anticancer [49]. In particular, some stilbene analogues have been synthesized to improve anticancer activity, developing some inhibitors of aromatase enzyme. These studies, mentioned above, underline the importance of the natural derivatives of stilbene for the therapy of different diseases [50].

**Figure 11:**
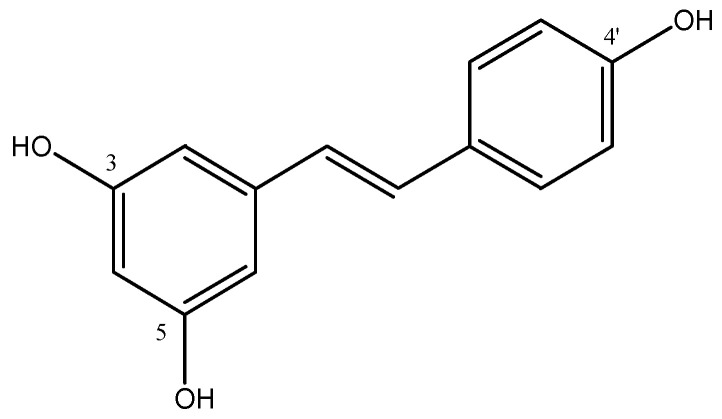
Chemical structures of resveratrol.

Resveratrol (RSV) is one of the most important stilbene derivatives studied (Figure 11).

RSV (3,4′,5-trihydroxy-*trans*-stilbene) is a phytoalexin, a well-known naturally occurring polyphenol that is a member of the stilbenoids subclass. This natural polyphenolic phytoalexin is present in grape skin and red wine and it is used for the therapy of cardiovascular disease. RSV and its derivatives show antioxidant [51] and anti-inflammatory activities against metabolic, cardiac, and neurodegenerative disorders [52]. RSV also showed cancer chemoprevention effects for its antioxidant activity [53]. The OH in 4′-position of RSV is important to eliminate free radicals, and its interaction with 4′-hydroxystyryl moiety and DNA polymerase showed an inhibition mechanism of cell cycle progression of cancer cells [54]. For these properties, many substituents were introduced on the aromatic rings of RSV with the aim to study some effects, for example, for pancreatic cancer [53]. Especially, RSV analogues were synthesized by modifying 3,5-OH phenyl with another aromatic ring, while the 4′-OH was not modified because it is important for the antioxidant activity [53]. These molecules showed a good cytotoxic activity in pancreatic cancer cell lines, becoming potential candidates as anticancer drugs and suggesting that 3,5-dihydroxy of RSV is not important for cytotoxic activity in these cell lines [55]. RSV has antioxidant and estrogenic activity and antagonist activity against aryl hydrocarbon receptor (AhR).

**Figure 12:**
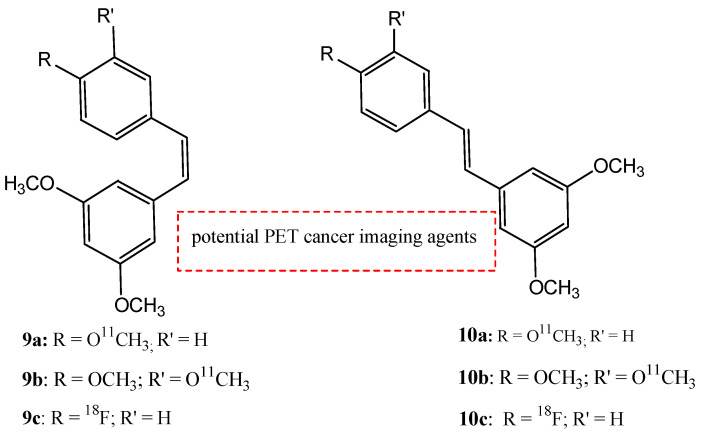
Chemical structures of radiolabeled stilbene derivates **9a**–**c** and **10a**–**c**.

AhR is an intracellular, ligand-dependent, transcription factor that modulates the expression of a lot of genes in many tissues and species [56]. Some stilbene derivates were labeled with carbon-11 and fluorine-18 to develop six potential targets to monitor AhR expression as PET cancer imaging agents [57]. The six stilbene derivates shown in Figure 12 are *cis*-3,5-dimethoxy-4′-[^11^C]methoxystilbene (**9a**), *cis*-3,4′,5-trimethoxy-3′-[^11^C]methoxystilbene (**9b**), *trans*-3,5-dimethoxy-4′-[^11^C]methoxystilbene (**9c**), *trans*-3,4′,5-trimethoxy-3′-[^11^C]methoxystilbene (**10a**), *cis*-3,5-dimethoxy-4′-[^18^F]fluorostilbene (**10b**), and *trans*-3,5-dimethoxy-4′-[^18^F]fluorostilbene (**10c**).

Compounds **9a**–**c** and **10a**–**c** are AhR antagonists with high receptor binding activity, with Ki = 75 ± 3.2 nM, 7.7 ± 0.2 nM, 96 ± 3.4 nM, and 3.1 ± 0.8 nM for compounds **9a**, **10a**, **9c**, and **10c**, respectively. Further studies are needed to evaluate in vivo biological tests for their use as new PET cancer AhR imaging agents [57].

**Figure 13:**
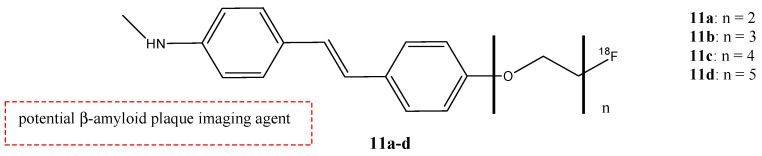
Chemical structures of ^18^F-Polyethyleneglycol stilbenes **11a**–**d**.

Zhang et al. analyzed the potential use of ^18^F-labeled polyethyleneglycol (PEG)-stilbene derivates **11a**–**d** as potential β-amiloid plaque imaging probes for PET (Figure 13) [58]. In these molecules, the radionuclide ^18^F is linked to the stilbene with a PEG chain with a range of ethoxy groups from two to five. The use of PEG groups in the stilbene chemical structure was important to lower the lipophilicity and improve bioavailability. These new fluorinated stilbene derivates showed an excellent binding affinity (Ki = 2.8–5.2 nM). The addition of two to five PEG units does not change the binding affinity. These stilbene derivates showed an appropriate range of lipophilicity (logP value was 2.52, 2.41, 2.05, and 2.28 for *n* = 2–5, respectively) and they penetrated the BBB with an excellent uptake in the brain of normal mice (6.6–8.1% dose/g brain) with a 2 min post-intravenous injection. Compounds **11a**–**d** have been studied in normal mouse brains, and they showed an initial uptake and a rapid wash out (1.2–2.6% dose/g brain) at 60 min post-intravenous injection. This feature is possible because in normal mouse brains, there are no β-amyloid plaques, and so these showed that these radiolabel agents could be used as β-amyloid plaques-targeting imaging agents. The PEG chain showed an efficient prosthetic group for ^18^F labeling. The fluorinated PEG stilbene derivates showed an excellent binding affinity towards Aβ plaques and also a good penetration of the BBB [58].

**Figure 14:**
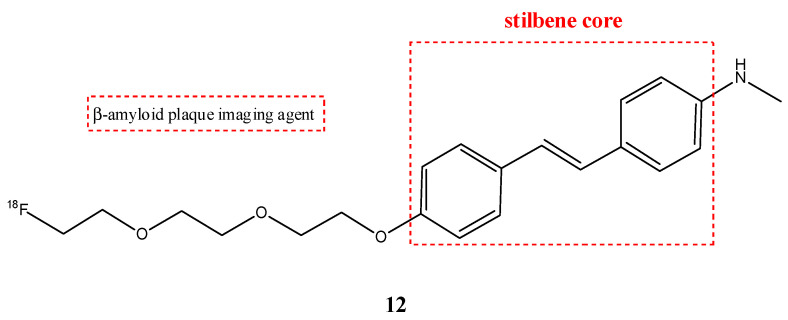
Chemical structure of florbetaben (**12**).

An important radiopharmaceutical ^18^F-labeled stilbene derivate is florbetaben (**12**) (also named ^18^F-BAY94-9172 or ^18^F-AV1/ZK and *trans*-4-(*N*-methylamino)-4′-{2-[2-(2-[^18^F]fluoro-ethoxy)-ethoxy]-ethoxy}-stilbene), which is used as a positron emission tomography tracer to discover β-amyloid plaques in brains with AD (Figure 14). Florbetaben showed a good high affinity and specificity in vitro for β-amyloid plaques [59]. In fact, the most common adverse reaction can be erythema, irritation, and pain [60]. Florbetaben (**12)** binds to plasma proteins and it is metabolized by CYP enzymes, especially the CYP4F2, which carries out *N*-demethylation, while the formation of polar metabolites was executed by CYP2J2 and CYP3A4. Florbetaben is safe and well tolerated; it is used to monitor the therapy of the AD [41] and is also studied for PET imaging in the diagnosis of cardiac amyloidosis [61]. It has a suitable pharmacokinetic: florbetaben is eliminated from plasma primarily via the hepatobiliary system, with a mean biological half-life of ~1 h. In total, 26–36% of the radioactivity that is injected is eliminated in urine up to 12 h post-injection [42].

The stilbene derivates, as radiopharmaceutical **12**, show a high brain uptake and bind to proteins and aggregates of molecular conformation with adjacent beta-sheet structures typical both in amyloid plaques and in myelin basic protein (MBP). Thereby, stilbene derivative radiopharmaceuticals could also be used as radiotracers for clinical studies in multiple sclerosis and other demyelinating diseases [62]. In a recent clinical study, Cassano Cassano et al. highlighted the central role of the use of florbetaben in PET imaging in the diagnostic process of patients with suspected cardiac amyloidosis. From this study, the use of florbetaben can also be used to explore all sites of amyloid deposits, and it showed a valid technique to also find asymptomatic tissues of amyloid deposition [63].

**Figure 15:**
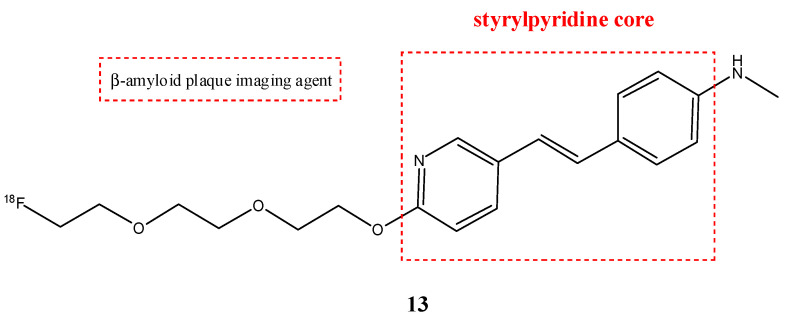
Chemical structure of florbetapir (**13**).

Florbetapir (**13**) (also called ^18^F-AV-45) is a radiopharmaceutical with a structure similar to florbetaben, with a modification of the stilbene core with a styrylpyridine moiety (Figure 15). This structural difference causes a lower lipophilicity of the molecule and shows faster brain kinetics compared with florbetaben [64]. This stilbene derivate radiopharmaceutical is also used as a PET tracer for the imaging of amyloid plaques in AD. Indeed, it showed high binding affinity and specific labeling of amyloid plaques in the cortical regions and hippocampus [65]. This radiopharmaceutical has a good ability to cross the BBB and has rapid kinetics. Preclinical studies showed that it has a quick cleaning from the blood circulation [66]. Recently, for the first time, a clinical study demonstrated that, in systemic light-chain amyloidosis, florbetapir PET/CT could detect early right ventricular (RV) amyloid before changes to RV structure and function occur. This study showed that RV dysfunction and poorer RV structure and function are all predicted by elevated RV amyloid on florbetapir PET/CT. These findings suggest that RV amyloid plays a key role in the pathophysiology of RV dysfunction and that florbetapir can also be used in the diagnosis of cardiac amyloidosis as florbetaben [67].

**Figure 16:**
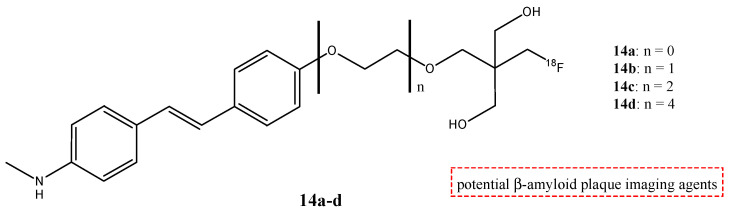
Chemical structures of stilbene derivates with ^18^F-fluorinated neopentyl glycol side chain **14a**–**d**.

Four ^18^F fluorinated stilbene derivates (**14a**–**d**) were analyzed, adding a neopentyl glycol side chain (NGS) (Figure 16) [68]. These molecules could show their potential use as compounds for PET imaging of β-amyloid in Alzheimer’s disease. The stilbene moiety was used and chosen because it has a relatively planar conformation that is important for binding β-amyloid, and the introduction of the NGS group seemed to have a better effect on the affinity.

From the results of the pharmacokinetic studies of these compounds in normal mice, compound **14a** (with *n* = 0) nonpegylated showed a relatively slow blood clearance compared to the other pegylated neopentyl compounds, and the brain uptake peaked at 10 min post-injection. Compounds **14b** (with *n* = 1) and **14c** (with *n* = 2) showed a peak brain uptake at 2 min post-injection followed by fast clearance; instead, compound **14d** showed a lower initial brain uptake than the other compounds, and this result suggested a poor BBB permeability. From this study, ^18^F fluorinated stilbene derivates with a neopentyl glycol side chain with two, one, or no PEG linkers (compounds **14a**–**c**) demonstrated a high initial uptake and a rapid clearance from the brain and blood in normal mice, which are the important features of ideal β-amyloid radioligands to reduce the background radioactivity [68].

**Figure 17:**
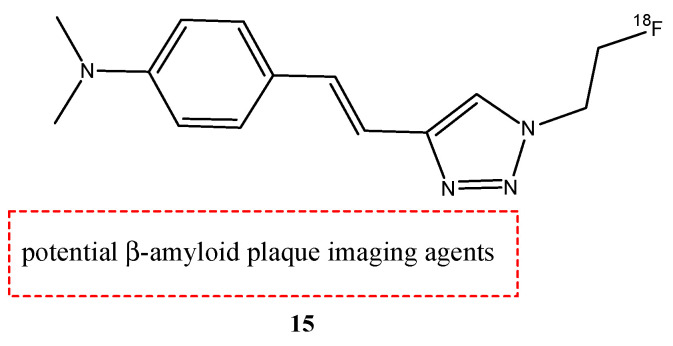
Chemical structure of ^18^F-labeled styryltriazole **15**.

**Figure 18:**
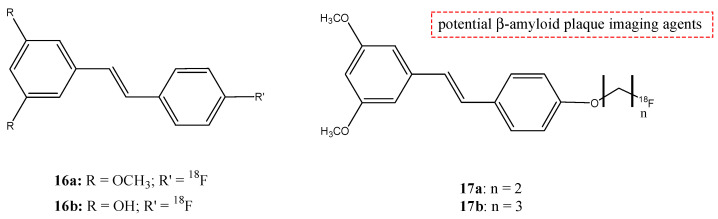
Chemical structure of ^18^F-labeled resveratrol derivates **16a**,**b** and **17a**,**b**.

Lee et al. synthesized and studied the biodistribution of ^18^F-labeled styryltriazole (**15**) (Figure 17) and four resveratrol derivates, **16a**,**b** and **17a**,**b** (Figure 18), to study their potential use for Aβ plaque imaging [69].

The fluoroethyl (**17a**) and fluoropropyl resveratrol derivates (**17b**) showed an excellent binding affinity (Ki = 0.74 nM and Ki = 049 nM). The *O*-demethylation at the 3,5-positions of compound **16b** (Ki = 39.7 nM) decreased the binding affinity with respect to compound **16a** derivate (Ki = 4.91 nM). This suggests that methoxy groups at the 3,5-positions are important to have a high binding affinity to β-amyloid (1–42) aggregates. Furthermore, the styryltriazole derivative **15** showed a great binding affinity to β-amyloid (1–42) aggregates (Ki = 12.8 nM). From these results, compounds **15** and **17b** were chosen to radiolabel with ^18^F: compound **15** was chosen because it has a triazole moiety and showed a lower binding affinity than resveratrol derivates **16a**, **17a,** and **17b**; and compound **17b** was chosen because it showed the highest binding affinity value. The partition coefficients of these two molecules [^18^F]**15** and [^18^F]**17b** were studied, and their values of log *p* were 1.74 and 2.84, respectively. These results suggest a favorable brain permeability. From these results, [^18^F]**15** could be a potential radioligand for β-amyloid plaque imaging, because it showed a high initial uptake and a fast wash-out from normal mouse brains with respect to [^18^F]**17b,** which showed slow pharmacokinetics in normal mouse brains with metabolic defluorination, despite [^18^F]**17b** showed the highest binding affinity to β-amyloid (1–42) aggregates [69].

**Figure 19:**
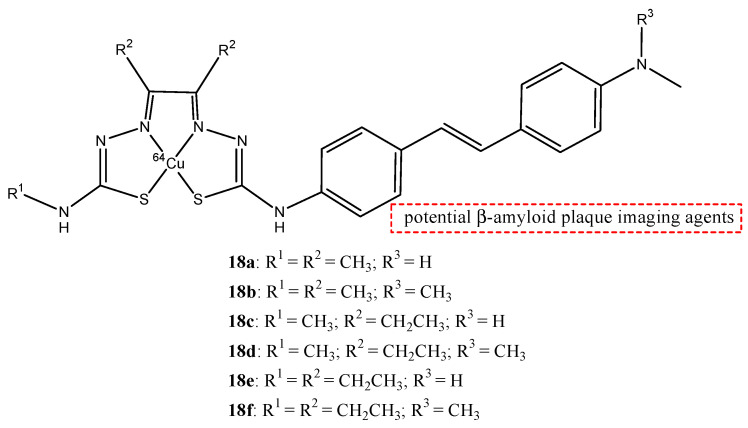
Chemical structures of ^64^Cu-labeled Bis(thiosemicarbazonato)-stilbenyl complexes **18a**–**f**.

Noor et al. analyzed the use of the radionuclide copper-64 (^64^Cu) to form some stilbene derivative complexes (**18a**–**f**), as shown in Figure 19 [68]. Copper-64 is a positron-emitting radionuclide that has a radioactive half-life of 12.7 h, and it is easy to transport it to facilities remote to the production site. They studied the bis(thiosemicarbazonato)-stylbenyl complexes of copper-64 **18a**–**f** to have potential radioprobes that bind to amyloid-β plaques for diagnosis with imaging of AD. Tetradentate bis(thiosemicarbazonato) was conjugated to stilbene functional groups to form charge-neutral and lipophilic complexes with copper radionuclides.

The substituents on the backbone of bis(thiosemicarbazonato) copper II complexes can modify the lipophilicity, membrane permeability, and retention to alter noncovalent interactions with serum proteins. The electron-donating substituents, such as -NHCH_3_ or N(CH_3_)_2_, help the stilbene derivates to bind the β-amyloid plaques. The lipophilicity of these complexes was studied by measuring their octanol-buffer distribution coefficients (log D_7.4_) to evaluate their possibility to cross the BBB through passive diffusion. The compounds that cross the BBB have a log D in the range 1–3. All six complexes, **18a**–**f,** have a log D_7.4_ in the range 1.4–1.6, but **18b** showed a better brain uptake in wild type mice. The uptake was analyzed and expressed as a percentage of the injected activity normalized to the mass of the organ (% IA/g) and **18b** showed a good brain uptake after 2 min post-injection of 2.2 ± 0.6% IA/g [70].

## 4. Other Natural Compound Derivates

### 4.1. Benzofuran Derivates

**Figure 20:**
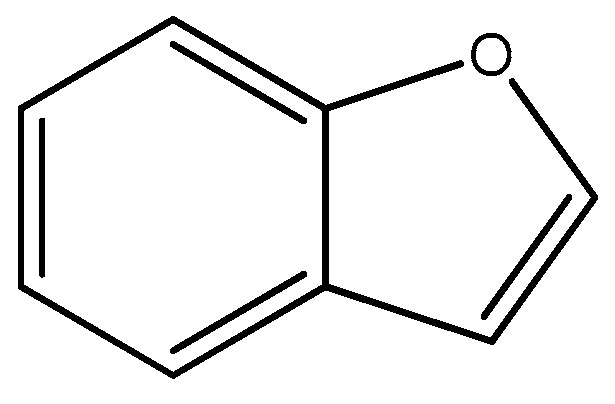
Chemical structure of benzofuran core.

Benzofuran is an important core (Figure 20) that is present in many natural derivates used in many therapeutic areas such as cancer, inflammation [71,72], hormonal imbalance, renal disorders, and cardiovascular diseases [73]. Benzofuran derivates have also antipyretic, anticoagulant, and analgesic properties [74].

These molecules are important for diabetes mellitus, because benzofuran derivates are active against a lot of targets of these diseases, such as PTP1B, GPCR-40, glucokinase, and α-glucosidase [51]. Some neurological disorders, like Alzheimer’s, Parkinson’s, depression, drug abuse, memory dysfunction, memory loss, migraine, autonomic nervous system dysfunction, and spinal trauma, are treated with benzofuran derivatives. For these reasons, benzofuran derivates were studied to discover new possible radiotracers and radioligands for the diagnosis of a lot of diseases in nuclear medicine with PET or SPECT.

**Figure 21:**
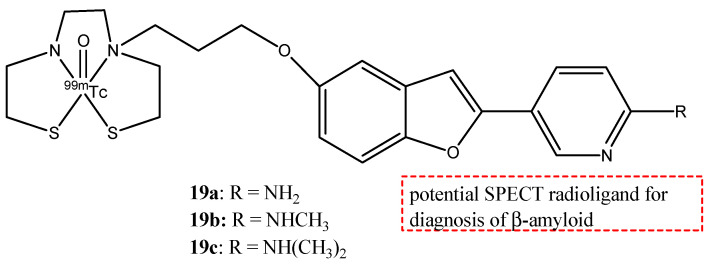
Chemical structures of ^99m^Tc-pyridyl benzofuran derivates **19a**–**c**.

With regard to neurological diseases, three novel benzofuran derivates, **19a**–**c** (Figure 21), were studied as potential probes for imaging for AD [74]. These radio probes have the radionuclide ^99m^Tc (T_1/2_ = 6.01 h, 141 keV), which is a radioisotope used in nuclear medicine as γ-emitting for SPECT.

In these molecules, a chelating structure is important for the transition of ^99m^Tc. To ensure the permeability of the probes of BBB, bis(aminoethanethiol) (BAT) as chelating ligand was added. The pharmacokinetics of ^99m^Tc-labeled pyridyl benzofuran complexes in the brain were studied in normal mice. The optimal log *p* value of a molecule to cross the BBB ranged from 0.1 to 3.5. The log *p* values for **19a**–**c** were 0.68, 1.35, and 2.09, respectively, and these values indicated that these complexes should penetrate BBB. The three ^99m^Tc-labeled complexes showed a good uptake in the brain within 10 min post-injection, but compound **19b** showed the highest initial uptake at 2 min post-injection (1.80% ID/g). From these results, **19b** was chosen to study their binding to β-amyloid plaques in Tg2576 transgenic mice, and it showed a good binding affinity. Complex **19b** should be a potential SPECT radioligand for the diagnosis of β-amyloid in AD [75].

### 4.2. Chalcone Derivates

**Figure 22:**
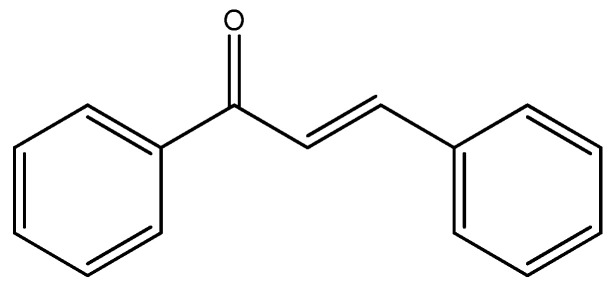
Chemical structure of chalcone.

Chalcone (1,3-diaryl-2-propen-1-one) (Figure 22) is a molecule with two aryl rings separated by α,β-unsaturated carbonyl group, and it is a chemical scaffold found in a lot of natural plants such as vegetables, spices, teas, and fruits [76]. Chalcone has a conjugated structure; it has a high delocalization of the electrons and it has many possibilities for undergoing electron transfer reactions [77].

**Figure 23:**
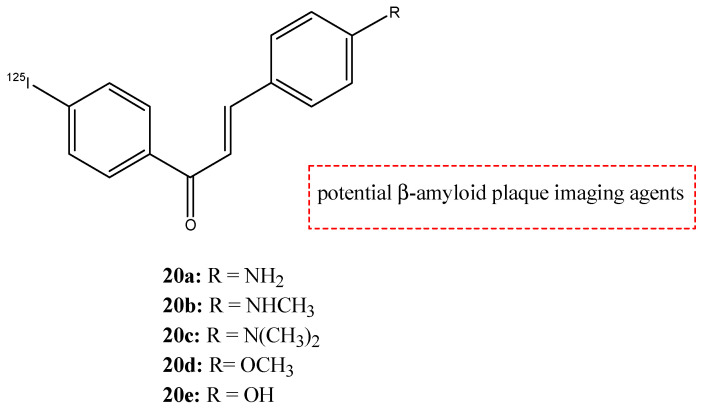
Chemical structures of [^125^I]-labeled chalcone derivatives **20a**–**e**.

The chalcones are precursors for flavonoids and isoflavonoids. These phytochemicals are available and nontoxic, and they are studied for their many biological activities, such as anticancer [78], anti-inflammatory [79], antidiabetic [80], cancer chemopreventive [81], antioxidant [82], antimicrobial [83], antileishmanial [84], and antimalarial activity [85]. Moreover, chalcone derivatives are molecules that showed a lot of pharmacological effects for the treatment of many diseases [45,53,86,87]. With regard to AD, chalcones and their derivatives showed an important role in inhibiting the β-amyloid fibrils aggregation [88]. For this potential activity to interact with β-amyloid plaques, chalcone derivates can be studied to develop potential radiolabeled probes for AD imaging. Thereby, these molecules need structural and chemical modifications to allow their radiolabeling with some probes used in nuclear medicine. A radiopharmaceutical for AD should have some features, including high BBB permeation, minimal off-target binding, rapid absorption, sudden washout from brain tissues, and low toxicity. Five chalcone derivatives, **20a**–**e,** containing an electron-donating group in position 4 of the first aromatic ring and an iodine-125 atom in position 4′ of the second aromatic ring (Figure 23), were studied [77].

Their binding affinity to synthetic β-amyloid (1–42) aggregates was studied, and the compound with dimethylamino moiety **20c** showed the highest specificity (K_d_ = 4.2 ± 1.1 nm). Additionally, their biodistributions were studied, and compounds **20a**–**e** displayed a high brain uptake ranging from 2.0 to 4.7% ID/g at 2 min post-injection and a good clearance.

**Figure 24:**
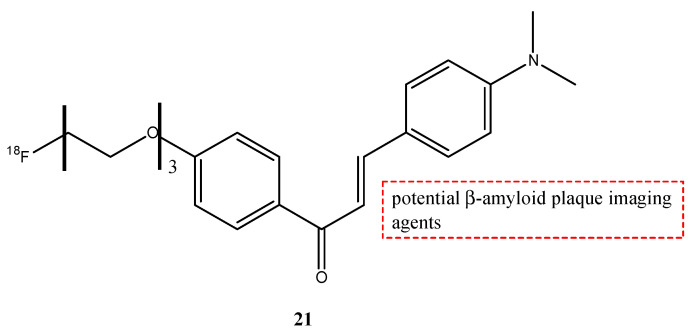
Chemical structure of [^18^F]-labeled chalcone derivative **21**.

Another [^18^F]labeled chalcone derivative **21** (Figure 24) was synthesized with the substitution at the amino group in position 4 with dimethylamino group, while the length of PEGylated (*n* = 3 PEG) chain in position 4′ was added [77]. This molecule showed a high uptake in normal mouse brain (3.85% ID/g) at 2 min post-injection and was cleared rapidly in 60 min. Finally, using Tg2576 transgenic mice and AD patients, the probe **21** was able to stain Aβ plaques.

**Figure 25:**
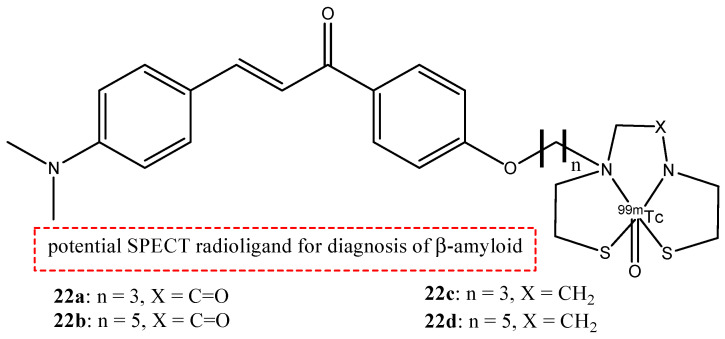
Chemical structures of [^99m^Tc]-labeled MAMA and BAT-chalcone derivatives **22a**–**d**.

Chalcone derivatives can also be labeled with radionuclide ^99m^Tc. This radionuclide is a metal, so it can be inserted in a chalcone derivative through a complexation reaction with chelators covalently linked to a molecular vector. The chelators are important elements because they can influence the molecular weight and the lipophilicity. These two features regulate the crossing of the BBB. Four chalcone derivatives (**22a**–**d**) were synthesized and studied [77,89]; they contain monoamine-monoamide-dithiol (MAMA) or bis-amino-bis-thiol (BAT) chelators linked to the backbones by a three- or five-atom-length alkyl linker (Figure 25).

**Figure 26:**
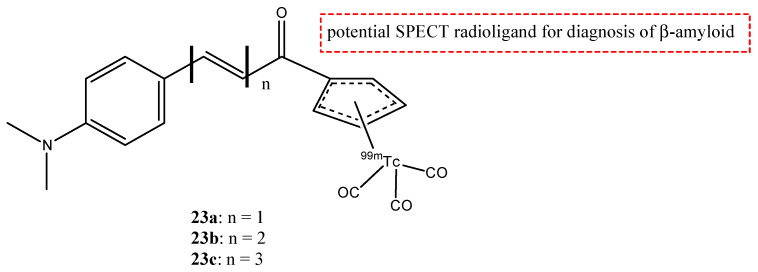
Chemical structures of [Cp^99m^Tc(CO)_3_]-chalcone mimic derivatives **23a**–**c**.

Biodistribution studies in normal mice showed that compound **22c** exhibited a high brain uptake (1.48% ID/g at 2 min post-injection) and a rapid elimination (0.17% ID/g at 60 min post-injection), while **22d**, **22b,** and **22a** showed poor initial uptake (from 0.32 to 0.78% ID/g at 2 min post-injection) and were cleared from the mice brain relatively slowly (0.11–0.16% ID/g at 60 min post-injection). From these results, the uptake and the pharmacokinetics of compound **22c** are better than the other [^99m^Tc]-labeled BAT and MAMA-chalcone derivatives, and compound **22c** could be studied and investigated as a potential radiotracer for β-amyloid imaging. These molecules, **22a**–**d,** were synthesized, generally, with a bifunctional approach (the presence of a chelator linked with a covalent linker to the vector molecule). But this approach causes an increase in the weight and the volume of the molecule, leading to some problems regarding the crossing of the BBB. For this reason, an integrated approach was used, and the replacement of parts of the targeting vector with the chelation moiety enables a minimal change in the weight, size, conformation, affinity, and planarity of the potential radiotracer. With regard to these premises, some chalcone complexes (**23a**–**c**) were also synthesized and studied with a [^99m^Tc]-tricarbonyl-cyclopentadienyl core ([Cp^99m^Tc(CO)_3_]) (Figure 26) [77].

The researchers showed that the distortion of planar configuration weakens; thus, the binding affinity for amyloid plaques is better. Biodistribution studies were carried out, and compound **23a** complex, with the shortest π-conjugation (*n* = 1), showed the highest initial brain uptake (4.10% ID/g at 2 min post-injection). The integrated approach suggests the possibility to use the chalcone structure to develop useful [^99m^Tc]-labeled radiopharmaceuticals for AD imaging [77,90].

## 5. Conclusions

Molecules of natural origin have an important impact on the pharmacological field, as they have a wide chemical and structural diversity and exhibit many biological activities; they are capable of being excellent leads for the development of molecules for use in various diseases [91]. In this review, we wanted to highlight the development of radiopharmaceuticals using the radiolabeling of natural derivatives molecules with the aim to obtain new compounds with different pharmacological activities (Table 1), and to highlight the importance of researching new molecules for use in nuclear medicine by exploiting the potential of natural scaffolds. These radiotracers are particularly useful because, by exploiting the physiological nature of the natural scaffold, they are able to deliver the radionuclide to the area of interest to decay and exploit the radiation for diagnostic purposes.

There are encouraging results in the development of new radiopharmaceuticals derived from molecules of natural origin by making appropriate chemical modifications that can maintain the stability of the tagging of the molecule with the radionuclide so that the radiation can reach the area of interest. Radiochemists can refer to this work to continue the search for new radiopharmaceuticals that provide high specificity, high binding affinity, low toxicity, high stability in the blood, and appropriate clearance.

## Figures and Tables

**Table 1:** Summary of the general structures of the radiolabeled compounds containing natural products and their main activity.

Compounds	Structures	Radionuclides	Pharmacological Effects	References
**1a**–**c**	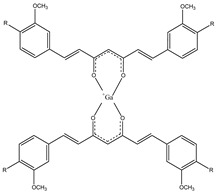	^68^Ga	Potential use for imaging of β-amyloid plaque and cancer.	[32]
**2**	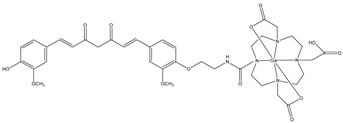	^68^Ga	Potential use of colon rectal cancer.	[34]
**3**	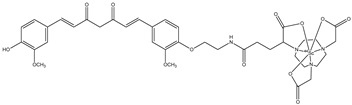	^44^Sc	Potential PET agent.	[30]
**4**	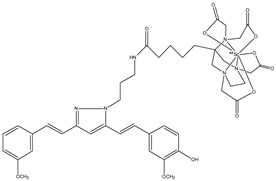	^44^Sc	Potential PET agent.	[30]
**5**	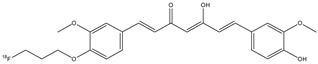	^18^F	Potential diagnostic use for Alzheimer’s disease.	[35]
**6**	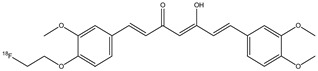	^18^F	Potential diagnostic use for Alzheimer’s disease.	[29]
**7**	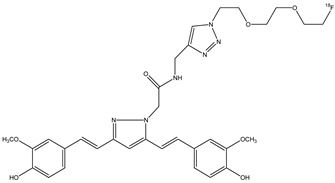	^18^F	Potential diagnostic use for Alzheimer’s disease.	[33]
**8a**,**b**	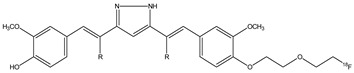	^18^F	Potential diagnostic use for glioma cancer.	[38]
**9a**–**c**	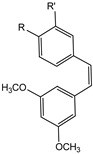	^18^F	Potential PET cancer imaging agents.	[57]
**10a**–**c**	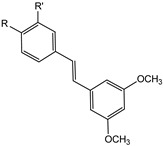	^18^F	Potential PET cancer imaging agents.	[57]
**11a**–**d**	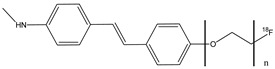	^18^F	Potential β-amyloid plaque imaging agent.	[58]
**12**	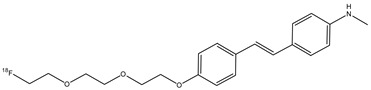	^18^F	β-amyloid plaque imaging agent.	[59]
**13**	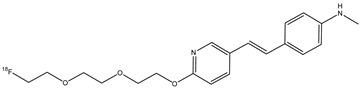	^18^F	β-amyloid plaque imaging agent	[65]
**14a**–**d**	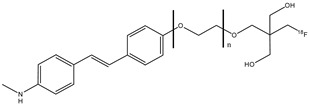	^18^F	Potential β-amyloid plaque imaging agents.	[68]
**15**	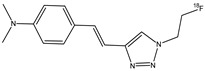	^18^F	Potential β-amyloid plaque imaging agents.	[69]
**16a**,**b**	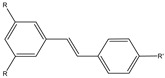	^18^F	Potential β-amyloid plaque imaging agents.	[69]
**17a**,**b**	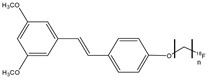	^18^F	Potential β-amyloid plaque imaging agents.	[69]
**18a**–**f**	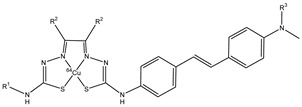	^64^Cu	Potential β-amyloid plaque imaging agents.	[70]
**19a**–**c**	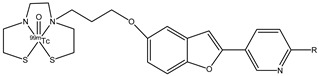	^99m^Tc	Potential SPECT radioligand for diagnosis of β-amyloid.	[75]
**20a**–**e**	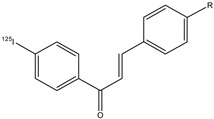	^125^I	Potential β-amyloid plaque imaging agents.	[77]
**21**	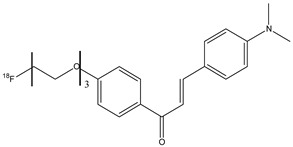	^18^F	Potential β-amyloid plaque imaging agents.	[77]
**22a**–**d**	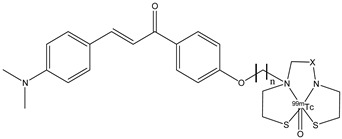	^99m^Tc	Potential SPECT radioligand for diagnosis of β-amyloid.	[77]
**23a**–**c**	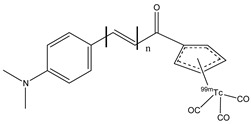	^99m^Tc	Potential SPECT radioligand for diagnosis of β-amyloid.	[77]

## Data Availability

Not applicable.
